# National Trends of the 5Ms of Geriatric Medicine, NHANES 2001-2018

**DOI:** 10.1093/geroni/igaf122.500

**Published:** 2025-12-31

**Authors:** Lily Bessette, Linnea Wilson, Timothy Anderson

**Affiliations:** University of Pittsburgh, Pittsburgh, Pennsylvania, United States; Beth Israel Deaconess Medical Center, Boston, Massachusetts, United States; University of Pittsburgh, Pittsburgh, Pennsylvania, United States

## Abstract

Age-friendly care of older adults embodies the Geriatric-5Ms, a framework spanning domains: Mind, Mobility, Medications, Multi-complexity, and Matters most. How the prevalence of these 5 geriatric syndromes is changing among U.S. adults is unknown. We analyzed cross-sectional data from the National Health and Nutrition Examination Survey (NHANES) using sampling weights to generate nationally representative estimates of the 5Ms in U.S. adults from 2001-2018. The geriatric syndromes (i.e. 5Ms) were defined by self-report of depression or cognitive impairment (Mind), functional impairments (Mobility), polypharmacy (Medications), >2 chronic conditions (Multi-complexity), and self-reported poor health (Matters Most). We estimated the prevalence of each of the 5Ms and performed age-stratified and sex-adjusted logistic regression to assess temporal trends. The cohort included 47,954 U.S. adults (38% younger, age 20-39; 45% middle-aged, age 40-64; 18% older, age ≥65; 52% female; 68% White). Overall, the prevalence of having at least one geriatric syndrome increased from 62% to 69% between 2001-2018, with greatest increases in the Mobility and Multi-complexity domains. In older adults, prevalence of Multi-complexity was highest of the 5Ms (93%), while the Medication domain increased the most over time (29% to 44%). In both middle-aged and older adults, the prevalence is increasing at similar rates in Mobility (middle-aged: 23% to 32%; older: 70% to 77%) and Multi-complexity (middle-aged: 63% to 70%; older: 85% to 93%) domains. Two-thirds of U.S. adults have at least one geriatric syndrome. The burden of the 5Ms is growing in middle-aged and older adults suggesting a need for age-friendly care across the lifespan.

